# Transposon Silencing Keeps Jumping Genes in Their Place

**DOI:** 10.1371/journal.pbio.0040353

**Published:** 2006-09-12

**Authors:** Liza Gross

Nearly a century ago, two geneticists described “rogue” pea plants with an unorthodox pattern of inheritance. William Bateson and Caroline Pellew found that crossing inferior rogues with normal plants always produced rogue offspring, suggesting that the rogue appearance was a dominant trait. The real surprise came when rogue progeny were crossed back to normal plants. Following the principles of Mendelian inheritance, these crosses should have produced a mix of normal and rogue plants, but they produced only rogue plants. The phenomenon, later dubbed “paramutation,” allowed the rogues to break the rules by acting “epigenetically”—inducing heritable changes in gene expression without DNA mutations. In one-sided interactions between gene pairs, or alleles, only “paramutagenic” alleles can attenuate, and eventually silence, the expression of “paramutable” alleles.

Epigenetic silencing involves chemical modifications to DNA and the histone proteins that remodel the chromatin surrounding DNA, rendering genes inaccessible to transcription-related proteins. Epigenetic silencing also targets “transposons,” genetic elements that can jump around the genome. Both paramutagenic alleles and transposons contain tandem or inverted repetitive DNA sequences. Recent work in a variety of species suggests that such repeats can induce heritable silencing when they trigger the production of double-stranded RNAs, which are then processed into small interfering RNAs that can inactivate genes through DNA methylation and other mechanisms.

A new study by Margaret Roth Woodhouse, Michael Freeling, and Damon Lisch sheds light on these somewhat mystifying processes by identifying a gene that keeps both transposons and paramutated color genes silenced in maize, confirming results recently published in *Nature*. The gene, *Mediator of paramutation1 (Mop1)*, encodes an RNA-processing enzyme called RNA-dependent RNA polymerase 2 (RDR2) that is required to make the small RNAs needed to maintain silencing of a transposon (called *MuDR*). While *Mop1* maintains *MuDR* silencing, the authors show that a second gene is required to establish heritability of silencing.

*MuDR* can be heritably silenced by a paramutagenic gene called *Mu killer (Muk)*—an inverted repeat variant of the *MuDR* transposon. *MuDR* encodes two genes—*mudrA* causes excision of the element and, with *mudrA*, *mudrB* helps reinsert it. *Muk* produces hairpin double-stranded RNAs that trigger rapid processing of full-length *mudrA* into small RNAs, leading to the destruction of *mudrA* transcripts and the methylation and silencing of *MuDR*. *Muk*-induced *MuDR* silencing begins with *mudrA*, then spreads to the adjacent *mudrB* gene.

Mutations in *Mop1* interfere with both *Mutator* transposon methylation and paramutation of several maize color genes. Woodhouse et al. found that *mop1* is an evolutionary cousin of the RDR2 found in *Arabidopsis*, where it effects transposon silencing. Maize plants carrying two mutant copies of *mop1* failed to produce small RNAs corresponding to *mudrA* and *mudrB*—confirming *Mop1*’s role as an RNA-processing enzyme.

**Figure:**
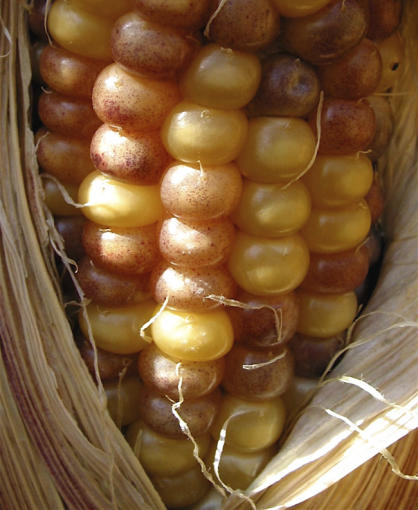
Heavy spotting on corn kernels reveals the activity of the *Mutator* system. (Photo: Damon Lisch)

To test the *mop1* mutant’s effects on *Muk*-induced *MuDR* silencing, the researchers bred plants that carried both *Muk* and *MuDR* in the presence or absence of a functional copy of the *Mop1* gene. Mutant plants showed clear evidence of *MuDR* silencing, suggesting that *Mop1* is not required to initiate silencing. These plants also continued to produce small RNAs specifically associated with the initiation of silencing. Thus, although *Mop1* is required to make the small RNAs associated with the maintenance of *mudrA* and *mudrB* silencing, it is not required to make the small RNAs associated with the initiation of *Muk*-induced *MuDR* silencing. Further, the progeny of these mutant plants carried only inactive *MuDR* elements, indicating that *Mop1* is not required for heritable silencing of *MuDR*—though there appear to be other factors that are required for this process. Offspring of plants that had both *MuDR* and *Muk* but lacked functional nucleosome assembly protein 1 (NAP1, a chromatin-building protein) gave rise to heavily spotted kernels—the sign that heritable *MuDR* silencing had been disrupted. Yet the loss of NAP1 did not block the *initiation* of silencing, because these plants had methylated *Mutator* elements whether or not they expressed NAP1, which suggests that *mudrA* activity had been lost in both cases. Thus, while losing NAP1 didn’t prevent *Muk* from initiating *MuDR* silencing, it did prevent *Muk* from establishing a stable, heritably silenced state.

Altogether, these results show that distinct factors initiate, establish, and maintain *MuDR* silencing. *Muk* initiates silencing by targeting *mudrA* with its hairpin RNAs, leading to the destruction of mudrA transcripts and methylation of the transposon’s terminal inverted repeats. NAP1 is required to establish heritable silencing, likely by changing chromatin into a transcription-unfriendly state. Mop1/RDR2 then maintains silencing by using RNA processing to mediate continued DNA methylation.

Given the damage that transposons can cause by inserting themselves into essential genes, it’s not surprising that organisms have evolved enduring mechanisms to keep jumping genes in their place. This study contributes a valuable framework for identifying the factors that regulate the enigmatic epigenetic processes that defend the genome against invasive elements—and helps explain how these changes can persist and be transmitted to the next generation.

